# Effect of Sanxan Gum on the Properties of Rice Flour-Based Coating Batter and the Quality of Deep-Fried Battered Pork Slices

**DOI:** 10.3390/foods15213900

**Published:** 2026-10-30

**Authors:** Xiaolong He, Junxian Song, Jiahui Liao, Xianghong Zhang, Zhongyang Dong, Xiangli Ding, Weiming Fang, Jianhua Tang

**Affiliations:** 1 School of Tourism and Culinary Science, Yangzhou University, Yangzhou 225009, China; 2 Key Laboratory of Chinese Cuisine Intangible Cultural Heritage Technology Inheritance, Ministry of Culture and Tourism, Yangzhou University, Yangzhou 225009, China

**Keywords:** sanxan gum, rice flour-based batter, deep-fat frying, oil reduction, gel network, microstructure

## Abstract

In fried food systems, the absence of gluten in rice flour-based coating batters results in low viscosity and poor structural integrity, thereby constraining their application. In this study, sanxan gum was incorporated into a rice flour-based batter to compensate for the viscosity deficiency, construct a denser gel network, and improve batter handling properties and oil reduction. The hydration properties, pasting behaviour, rheological characteristics, water mobility, and quality attributes of the fried products were evaluated. Results showed that sanxan gum increased batter viscosity and pick-up in a concentration-dependent manner and restricted starch granule swelling through competitive hydration. LF-NMR revealed no significant changes in water mobility, indicating that the oil-reducing effect of sanxan gum did not rely on water-state modulation. At 0.6% addition, the crust oil content (21.33%) decreased, meat tenderness (25.11 N) and moisture content (56.19%) improved, and the crust exhibited a continuous and homogeneous gel network. At 0.8% addition, although the crust oil content (15.07%) was the lowest, the total oil content per piece (0.69 g) was equivalent to that at 0.6% (0.70 g) due to moisture dilution and excessive crust thickness, accompanied by deteriorated microstructure and no further improvement in meat tenderness (24.05 N). These results demonstrate that sanxan gum effectively improves the functionality of rice flour-based coating batters and the quality of fried products by increasing viscosity, restricting starch swelling, and forming a continuous gel network, whereas excessive addition introduces structural drawbacks. This work provides a scientific basis for the application of sanxan gum as a functional hydrocolloid in deep-fried batter-coated food formulations.

## 1. Introduction

Deep-fat frying is a widely employed processing technique. However, it generates products with high oil content and has been associated with adverse health outcomes. Batter coating acts as a mass-transfer barrier that prevents moisture loss and reduces oil absorption while ensuring a juicy interior and crispy exterior. Hydrocolloids can further control batter viscosity, pick-up, and water-holding capacity to form a network that enhances this barrier effect, thereby minimizing mass transfer during frying. Accordingly, the incorporation of hydrocolloids into batter formulations has emerged as a viable oil-reduction strategy owing to its operational simplicity and compatibility with existing production processes [1]. Xanthan gum and guar gum represent the most extensively studied hydrocolloids for this application, and their incorporation into batters has been shown to reduce oil content by up to 53% compared with control samples, with xanthan gum alone lowering oil uptake by approximately 40% in fried snacks. Their efficacy is ascribed to enhanced batter viscosity and water-binding capacity, which restrict moisture loss and consequently impede oil penetration. However, the performance of these hydrocolloids is constrained by their thermal stability under high-temperature, high-oil frying conditions and by their dependence on the food matrix and processing parameters [1].

Sanxan, a novel high-acyl microbial polysaccharide produced by *Sphingomonas sanxanigenens* NX02, is a heteropolysaccharide composed of repeating tetrasaccharide units [2]. It contains 8.96% acetyl and 4.75% glyceroyl groups, classifying it as a high-acyl polymer. It forms a strong gel at concentrations above 0.1% and exhibits thermo-reversible gelation [3]. Furthermore, sanxan exhibits a pronounced synergistic thickening effect with modified corn starch, as the peak viscosity, trough viscosity, final viscosity, and pasting temperature of the mixed system increased with increasing sanxan concentration, and the microstructure of mixed gels showed a more homogeneous pore size distribution compared with starch gels alone [4]. These thickening and gelling properties render sanxan advantageous for constructing a dense, thermally stable gel network in batter systems during frying. Sanxan has also been applied in other food systems. In frozen dough, 0.5% sanxan improved fermentation capacity by protecting yeast viability and maintaining gluten network integrity during freeze–thaw cycles [5], and it reduced freezable water and inhibited ice recrystallization in frozen dough and steamed bread, thereby improving product quality [6]. Zhu et al. [7] further showed that sanxan regulated the gluten and starch matrix in frozen dough steamed bread and improved texture and sensory quality. In salt-free frozen cooked noodles, sanxan maintained texture during freeze–thaw cycles by controlling water and ice crystal status [8]. Sanxan also mitigated freeze–thaw deterioration of heat-denatured wheat gluten by stabilizing disulfide bonds and secondary structures [9]. In starch systems, sanxan modified the pasting and digestibility of *Cyperus esculentus* starch mixtures [10] and improved the pasting and microstructural properties of modified corn starch [4]. Its transparent hydrogel exhibits strong gelation and emulsifying properties [3]. These studies indicate that sanxan functions in diverse food matrices through water-holding, gel network formation, and freeze–thaw stabilization, but its use in batter systems remains unexplored.

However, research on hydrocolloids in batter applications has almost exclusively focused on wheat flour-based systems. Systematic investigations on rice flour-based batters remain scarce, as rice flour-based batters suffer from inherent deficiencies, including low viscosity, poor stability, and uneven coating due to the absence of gluten. Oh et al. [11] demonstrated that larger rice flour particle sizes resulted in low viscosity, uneven coatings, and high cooking loss. Shih and Daigle [12] reported that although rice flour resisted oil absorption better than wheat flour, it was less effective as a thickening agent. Due to the inferior water absorption capacity of rice flour, substituting more than 50% of wheat flour with rice flour results in unsuitable batter viscosity and stability [13]. Rahimi and Ngadi [14] reported that batters with high rice flour content exhibited decreased moisture, increased oil content, greater porosity, and a higher prevalence of surface holes and cracks after frying. These deficiencies severely limit the practical application of rice flour-based batters. Though hydrocolloids are generally effective at low concentrations (0.2–1.5% of dry batter ingredients), their performance is highly product-dependent and influenced by flour type [1]. To date, sanxan has not been explored in batter systems. In particular, its capacity to substantially increase viscosity at room temperature renders it particularly suitable for rice flour-based batters, which are characterized by low viscosity and instability. Therefore, simultaneously achieving viscosity regulation and oil-reduction enhancement in rice flour-based batters without relying on gluten proteins represents a critical scientific challenge.

Based on the unique characteristics of sanxan, sanxan gum was added to rice flour-based batter systems to compensate for the insufficient viscosity and loose structure caused by gluten deficiency while constructing a denser gel network to improve batter handling and coating properties and achieve significant oil reduction. The hydration properties, pasting behavior, rheological characteristics, water mobility, and fried product quality were evaluated to provide a scientific basis for the application of sanxan as a functional hydrocolloid in fried food formulations.

## 2. Materials and Methods

### 2.1. Materials

Sanxan gum (food grade, purity > 99%) was obtained from Hebei Xinhe Biochemical Co., Ltd. (Xingtai, China). Its purity was determined by gel permeation chromatography according to Huang et al. [2]. Rice flour (23.2% amylose) was bought from Xinxiang Liangrun Whole Grain Food Co., Ltd. (Xinxiang, China). Baking soda was purchased from Henan Boming Food Co., Ltd. (Xuchang, China). Frozen pork chop slices were purchased from Henan Shuanghui Investment & Development Co., Ltd. (Luohe, China). Refined soybean oil was bought from the Yihai Kerry Food Marketing Co., Ltd. (Yingkou, China).

### 2.2. Preparation of Composite Flour, Coating Batter, and Deep-Fried Meat

Rice flour was blended with baking soda and salt at a ratio of 100:1.0:3.0 (*w*/*w*/*w*). Sanxan was then added at different mass ratios based on rice flour (0%, 0.2%, 0.4%, 0.6%, and 0.8%, *w*/*w*). After mixing, all composite flours were sieved twice before use to obtain a uniform mixture. Coating batter was prepared by mixing composite flour with distilled water at a ratio of 1:1.15 (*w*/*v*), followed by stirring for 5 min at 150 rpm using a vertical mixer (JB-80SH, Shanghai Xiniu Laibo Instrument Co., Ltd., Shanghai, China) and standing for 5 min prior to use. Pork chop slices were thawed at 4 °C for 12 h and cut into uniform slices (4 cm × 4 cm × 0.5 cm) and then patted dry with paper towels to remove excess surface moisture prior to batter coating (approximately 9.5 g). For each treatment, the batter preparation and frying procedure were independently replicated three times. In each replication, fresh batter was prepared from 100 g of rice flour and used for five pork slices, with each slice fried individually in a separate batch. After five slices, the remaining batter was discarded, and fresh batter was prepared for the next replication to prevent moisture from the meat from entering the batter and altering its viscosity. The pork slices were completely immersed in the batter and drained for 10 s. For the batter without sanxan, which exhibited low viscosity, the draining time was reduced to 3 s to maintain adequate coating. Frying was performed in 3 L of soybean oil at 180 °C for 3 min with a commercial fryer (RJ-81, Guangzhou Aikai Catering Equipment Co., Ltd., Guangzhou, China). Only one slice was fried in each batch, and the slice was turned over every 20 s to ensure uniform browning. After frying, the samples were removed from the fryer and allowed to cool at room temperature for 10 min. The crust was carefully separated from the meat for further analysis.

### 2.3. Determination of Hydration Properties of Composite Flour

Water-holding capacity (WHC), oil-absorption capacity (OAC), water absorption index (WAI), water solubility index (WSI), and swelling power (SP) of composite flour were determined according to the method described before [15,16]. Briefly, 1.0 g of sample was mixed with 10 mL of distilled water (oil) in a centrifuge tube and vortexed for 30 s. After standing at room temperature for 30 min, the mixture was centrifuged at 3000× *g* for 20 min. The supernatant water (oil) was decanted, and the WHC (OAC) was expressed as the weight of water (oil) absorbed per gram of dry sample. A 1.0 g sample of composite flour was mixed with 10 mL distilled water. The mixture was vortexed and then incubated in a water bath at 30 °C for 30 min with intermittent stirring. The suspension was then centrifuged at 3000× *g* for 15 min. The sediment was weighed, and the WAI was calculated as the weight of the sediment divided by the weight of the dry sample. For WSI and SP, the mixture was cooled on ice for 10 min after being heated in a boiling water bath, then centrifuged at 3000 rpm for 10 min at 4 °C. The weight of the pellet and the solids remaining in the supernatant were used to calculate WSI and SP.

### 2.4. Pasting Properties

Rapid visco analyzer (RVA) analysis was conducted using an RVA-4500 instrument (Perten Instruments, Hägersten, Sweden) with the standard method [17]. The sample (3.0 g, dry basis) was mixed with 25 mL of distilled water in an RVA canister. The standard heating program was applied as follows: holding at 50 °C for 1 min, heating to 95 °C at 12 °C/min, holding at 95 °C for 2.5 min, cooling to 50 °C at 12 °C/min, and holding at 50 °C for 2 min. The pasting parameters, including peak viscosity, trough viscosity, breakdown viscosity, final viscosity, setback viscosity, and pasting temperature, were recorded.

### 2.5. Determination of Batter Characteristics

Low-field nuclear magnetic resonance (LF-NMR) analysis was performed using an NMI20-040V-I spectrometer (Suzhou Niumag Analytical Instrument Co., Ltd., Suzhou, China), equipped with a 25 mm probe. Approximately 5.0 g of batter was placed in the glass tube and inserted into the NMR probe. The transverse relaxation time (T_2_) was measured using the Carr-Purcell-Meiboom-Gill (CPMG) sequence with the following parameters: echo time (TE) of 0.3 ms, waiting time (TW) of 4000 ms, number of echoes (NECH) of 15,000, and number of scans (NS) of 4. The relaxation data were analyzed using MultiExp Inv Analysis software (Ver 4.0, Suzhou Niumag Analytical Instrument Co., Ltd., Suzhou, China) to obtain the T_2_ distribution and the proportions of different water populations.

Rheological measurements were performed using a Haake MARS III Rheometer (Thermo Scientific, Karlsruhe Co., Ltd., Oberhausen, Germany) according to the method of Ding et al. [16] with modifications. Approximately 5 g of batter was loaded onto the plate, and the gap between the plates was fixed at 1 mm. The sample was allowed to equilibrate for 5 min before testing. A frequency sweep test was conducted at 25 ± 1 °C in the linear viscoelastic limit (strain = 0.05%) under a constant stress of 10 Pa, with frequency varying from 0.1 to 10 Hz. The storage modulus (*G*′), loss modulus (G″), and tan *δ* were recorded as a function of frequency.

Differential scanning calorimetry (DSC) analysis was performed using a PerkinElmer DSC 6000 differential scanning calorimeter (PerkinElmer, Inc., Shelton, CT, USA) following the method of Zhu et al. [7] with modifications. Then, 20.0 mg batter was weighed into an aluminum pan and hermetically sealed. The samples were heated from 25 °C to 95 °C at a rate of 10 °C/min under a nitrogen atmosphere. An empty aluminum pan was used as the reference. The onset temperature (*T*_o_), peak temperature (*T*_p_), conclusion temperature (*T*_c_), and gelatinization enthalpy (Δ*H*) were recorded.

Apparent viscosity was measured using a rotational viscometer (NDJ-5S, Shanghai Lichen Bangxi Instrument Technology Co., Ltd., Shanghai, China) equipped with a No. 3 rotor at a rotational speed of 12 rpm. Batter pick-up was determined by weighing the pork slices before and after coating and was expressed as the percentage of the batter weight relative to the initial weight of the uncoated pork slice.

### 2.6. Quality Evaluation of Fried Samples

After frying, the color parameters of the fried crust were measured using a handheld colorimeter (CR-410, Konica Minolta (China) Co., Ltd., Beijing, China). The tenderness of meat was determined with a meat tenderness analyzer (C-LM3B, College of Engineering, Northeast Agricultural University, Harbin, China). Then, the crust was separated from the meat for analysis. The oil content of the crust was determined according to AOAC Official Method 960.39, while the moisture contents of both the crust and the meat were measured by the gravimetric method at 105 °C. The total oil content per fried piece (g/piece) was derived from the following mass balance equations, and the corresponding standard deviation was obtained via error propagation.

*m*_solids_ = the initial meat weight × batter pick-up × dry solid fraction in the coating batter (1)

*m*_crust_ = *m*_solids_/(1 − the moisture content of the crust − the oil content of the crust) (2)

The total oil per piece = *m*_crust_ × the oil content of the crust (3)

where *m*_solids_ is the mass of dry batter solids adhering to the meat (g), and *m*_crust_ is the total crust mass (g).

Crust samples were first defatted, then freeze-dried and sliced, followed by sputter-coating with a gold–palladium alloy. The microstructure of the crust was observed using SEM (Sigma 300, ZEISS, Oberkochen, Germany) under vacuum at an accelerating voltage of 5 kV. Representative micrographs were captured for each sample at ×50 and ×100 for the crust, respectively.

### 2.7. Statistical Analysis

Experiments were run in triplicate, and values are expressed as mean ± standard deviation. Data were statistically analyzed by one-way ANOVA. Treatment means were compared using Duncan’s multiple range test when ANOVA indicated a significant difference. Statistical significance was set at *p* < 0.05. Means labeled with different lowercase superscript letters are significantly different at *p* < 0.05. SPSS software version 26.0 (IBM Corp., Armonk, NY, USA) was used.

## 3. Results

### 3.1. Hydration Properties of Rice Flour with Various Addition Amount of Sanxan Guan

The hydration properties of rice flour were markedly affected by the amount of sanxan gum added (Table 1). While WHC increased significantly at 0.6% compared to lower addition amounts (0–0.4%), a significant increase in OAC was only observed at 0.8%. WAI and SP decreased at intermediate concentrations (0.2–0.4%) and then increased slightly at 0.8%. WSI exhibited an opposite trend, peaking at 0.4% and then declining at higher concentrations. The continuous enhancement of WHC and OAC can be attributed to the rigid entangled network formed by sanxan gum through hydrogen bonding, which physically entraps water and restricts its mobility, together with the hydrophobic acetyl and pyruvyl substituents that contribute to oil binding [10]. Similarly, Balangu seed gum increased batter WHC in a concentration-dependent manner, which was attributed to enhanced hydrogen bonding and electrostatic interactions between the polysaccharide and water molecules [18]. Similar concentration-dependent effects have also been reported for highland barley-wheat composite flour, where excessive crosslinking restricted swelling and led to constant or decreased WHC [15]. The non-monotonic changes in WAI, SP, and WSI indicate that low gum levels promoted amylose leaching into the continuous phase, whereas at higher levels the entangled network restricted water diffusion into starch granules and retained leached soluble components within the batter matrix. This is consistent with the reduced WSI and SP observed in highland barley-rice composite flours, where interactions among β-glucan, starch, and protein during thermal processing altered solubility and swelling behavior [16]. Similar effects have been reported for xanthan gum, which has been shown to decrease the swelling power and amylose leaching of rice starch as xanthan gum concentration increases [19,20]. At 0.8% sanxan gum, the slight increase in WAI and SP suggests that the swelling-restricting effect may have reached saturation or that the gum network underwent localized phase separation. These hydration changes would affect batter viscosity, coating pick-up, operability, and crust oil uptake, since increases in WHC and OAC raise batter viscosity and water retention, thereby improving coating pick-up and uniformity during dripping and alleviating the poor operability of low-viscosity rice flour batters. However, excessively high viscosity may lead to excessive dripping and a decline in coating pick-up. Moreover, although higher WHC improved moisture retention and reduced oil uptake, coating pick-up may decline when gum addition exceeds an optimal level [18]. A decrease in coating pick-up may counteract the oil-reducing effect of WHC and lead to higher oil uptake. As both WHC and coating pick-up are decisive factors for oil uptake [18], the oil-reducing effect of WHC can be fully expressed only when adequate coating pick-up is maintained. The intermediate reduction in WAI and SP, together with the decline in WSI at 0.6% gum, would favor the formation of a denser and more continuous starch–gum gel matrix with reduced porosity. This dense and continuous gel matrix would reduce pore formation and restrict oil migration during frying, consistent with the oil-barrier effect reported for hydrocolloids in fried breaded products [21]. Crust oil content was negatively correlated with WHC and positively correlated with WSI in highland barley–rice composite flour systems [16], and higher batter WHC was associated with greater moisture retention and lower oil uptake in fried chicken fillets [18]. Accordingly, 0.6% sanxan gum addition, characterized by relatively high WHC, reduced WSI, and restricted starch swelling, may be favourable for producing a cohesive coating that limits oil penetration. In contrast, the high WSI at 0.4% could weaken batter integrity and create channels for oil ingress, while the slight increase in WAI and SP at 0.8% may indicate a less homogeneous network, potentially causing uneven coating and higher oil retention.

### 3.2. Pasting Properties of Rice Flour with Various Addition Amount of Sanxan Guan 

The pasting properties of rice flour were markedly influenced by sanxan gum addition (Table 2). While peak viscosity showed a slight decrease without statistical significance as the sanxan gum addition amount increased, final viscosity and setback exhibited a general decline, accompanied by a pronounced reduction in breakdown, an increase in trough viscosity, and a slight prolongation of peak time. These changes indicate that sanxan gum restricted starch granule swelling and enhanced the thermal and shear stability of the starch paste. The reduced breakdown and setback further suggest that starch granules were less susceptible to disintegration during heating and had a lower tendency to retrograde upon cooling, thereby promoting the formation of a continuous and stable gel network while minimizing shrinkage and cracking of the crust during cooling. These effects can be explained by competitive hydration between sanxan gum and starch, as evidenced by the hydration properties discussed above. Specifically, the increase in WHC and OAC, together with the decrease in WSI and SP at intermediate sanxan gum addition amounts, indicates that the gum network restricted water availability for starch hydration and limited amylose leaching. Similar behavior has been reported for xanthan gum, which decreases the swelling power and amylose leaching of rice starch as gum concentration increases [20]. The prolonged peak time further supports a delayed development of peak viscosity and granule swelling, likely because the highly hydrophilic sanxan gum network reduced the water available for starch granule swelling. This is consistent with the general behavior of hydrocolloids that compete with starch for water and restrict granule swelling [22]. The synergistic thickening between sanxan gum and starch may also contribute to the increased paste stability [4].

The pasting behavior of starch is known to influence the oil content of fried products by affecting the structural stability and integrity of the starch gel matrix [23]. A lower breakdown and reduced setback, as achieved at intermediate sanxan gum addition, would promote the formation of a uniform and dense gel network with fewer structural defects, which could potentially reduce oil penetration during frying. In a recent study on highland barley-rice composite flours, it is confirmed that trough viscosity was negatively correlated with crust oil content, and pasting temperature was positively correlated with oil content, further supporting the role of pasting behavior in oil uptake regulation [16]. However, at excessive sanxan gum addition amounts, the continuously low levels of breakdown and setback, which exhibited no further significant reduction compared to the 0.6% addition level, may not improve oil-barrier performance, because other factors, such as excessive coating thickness, retarded moisture evaporation, and network inhomogeneity, may also exert a substantial influence.

### 3.3. Water Mobility of Coating Batter with Various Addition Amount of Sanxan Guan

Water mobility, rather than moisture content, plays a more critical role in oil absorption during deep-fat frying. Decreased water mobility in composite flour would impede water evaporation during deep frying and then decrease the oil content of the deep-fried meat [15]. Water-state regulation is a major route for oil reduction, but its effectiveness is matrix-dependent. Cassia gum, for example, increases the proportions of tightly and weakly bound water while reducing free water in wheat flour–tapioca starch batters, thereby limiting oil absorption [24]. In highland barley-wheat batters, however, the incompatibility between β-glucan and gluten causes phase separation and heterogeneous water distribution, promoting oil ingress. In contrast, in highland barley–rice batters, β-glucan is compatible with rice starch and improves water distribution homogeneity, thereby reducing oil content [25]. Other hydrocolloids such as xanthan gum and guar gum mainly increase viscosity to restrict water mobility [1,26].

**Figure 1:**
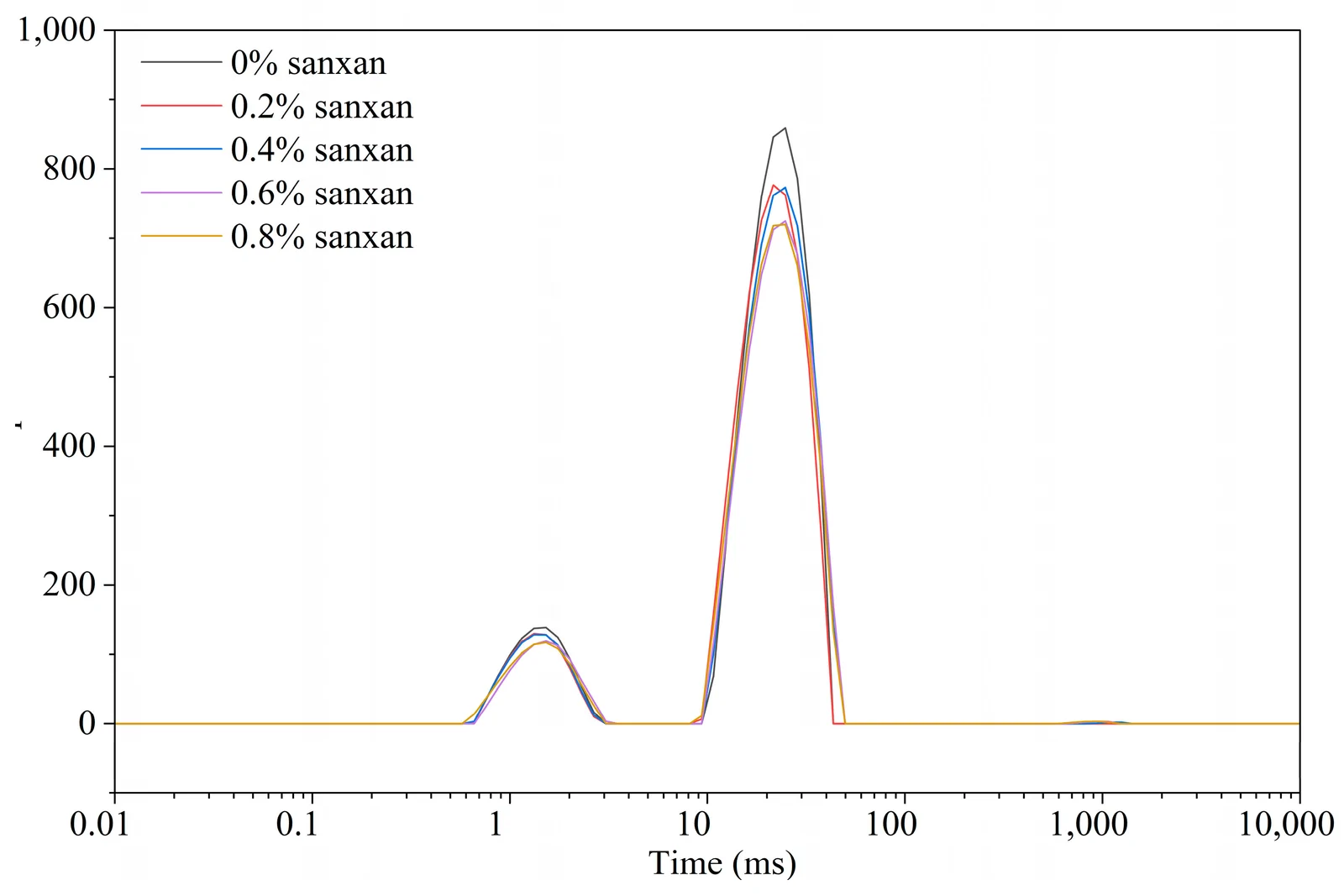
Effect of sanxan gum on water mobility of coating batter.

In wheat flour-based systems, sanxan can alter water states. Adding 0.5% sanxan to frozen dough increased strongly bound water and inhibited its transition to immobilized and free water during freeze–thaw cycles [6]. This finding was later confirmed in salt-free frozen cooked noodles [8]. In the present study, however, no statistically significant changes were detected in the LF-NMR parameters of rice flour-based batter (*p* > 0.05) (Figure 1). This discrepancy is driven by the matrix composition. In wheat flour systems, sanxan interacts with both gluten and starch, converting free water to bound water, whereas it interacts primarily with starch in rice flour systems. Sanxan is a high-acyl polymer that forms an elastic gel network through lateral association of double helices [17]. This network is established at room temperature and further reinforced during heating [6], and thus is present during batter preparation and persists throughout frying. Within this network, water molecules are physically entrapped via hydrogen bonding and steric confinement, which restricts their macroscopic diffusion but does not necessarily alter their local mobility within the network pores. Consequently, the transverse relaxation times and the relative proportions of water populations remain largely unchanged. This mechanism differs from that of hydrocolloids that convert free water into weakly bound water, which would alter T_2_ distributions. Furthermore, sanxan exhibits a synergistic thickening effect with starch, forming a homogeneous gel microstructure without phase separation [4]. This compatibility prevents water redistribution that often occurs when hydrocolloids and starch are thermodynamically incompatible. In the rice flour-based batter, the absence of gluten allows sanxan to interact directly with starch granules and form an interpenetrating network that uniformly confines water molecules. Therefore, instead of water-state modulation, the oil-reducing effect of sanxan in rice flour-based coating batter may involve interfacial barrier formation and gel network obstruction.

### 3.4. Starch Gelatinization Parameters of Coating Batter with Various Addition Amount of Sanxan Guan

The gelatinization temperatures (*T*_o_, *T*_p_, and *T*_c_) of the coating batters were essentially unchanged by sanxan addition. In contrast, the gelatinization enthalpy decreased at low sanxan concentrations and increased at higher concentrations. The reduction in Δ*H* at low concentrations (Table 3) indicates that less energy was required to melt the starch crystalline regions, which can be attributed to competitive hydration between sanxan and starch. The highly hydrophilic sanxan network formed at room temperature [3] reduces the water available for starch hydration and swelling. Although LF-NMR showed no significant change in overall water mobility, competitive hydration at low sanxan concentrations significantly restricts starch hydration and swelling. Similar reductions in Δ*H* have been reported for xanthan gum and curdlan, which also compete with starch for water [22]. The increase in Δ*H* at higher sanxan concentrations likely resulted from the denser and more interconnected sanxan network, which physically entrapped starch granules and restricted their swelling. In addition, the synergistic thickening between sanxan and starch through hydrogen bonding [4] reinforced the composite structure and increased the energy required for gelatinization. These two effects counterbalance each other, resulting in the observed non-monotonic Δ*H* trend. This thermal behavior contrasts with that of Cassia gum in a wheat flour-tapioca starch batter. Ran et al. [24] reported that Cassia gum increased *T*_p_ and Δ*H*, an effect attributed to its promotion of starch crystalline rearrangement. In contrast, sanxan, a high-acyl polymer, forms an elastic gel network through double-helix association [3] and interacts with starch mainly via physical entrapment rather than crystalline reinforcement. In the rice flour-based matrix, the absence of gluten allows sanxan to interact more directly with starch granules, which may amplify the water-competition effect at low concentrations. The lower Δ*H* at low sanxan concentrations suggests that less thermal energy was required to melt the starch crystalline regions, which may promote earlier formation of a continuous gel network during frying, contributing to the barrier properties of the coating against oil penetration.

### 3.5. Rheological Properties of Coating Batter with Various Addition Amount of Sanxan Guan

**Figure 2:**
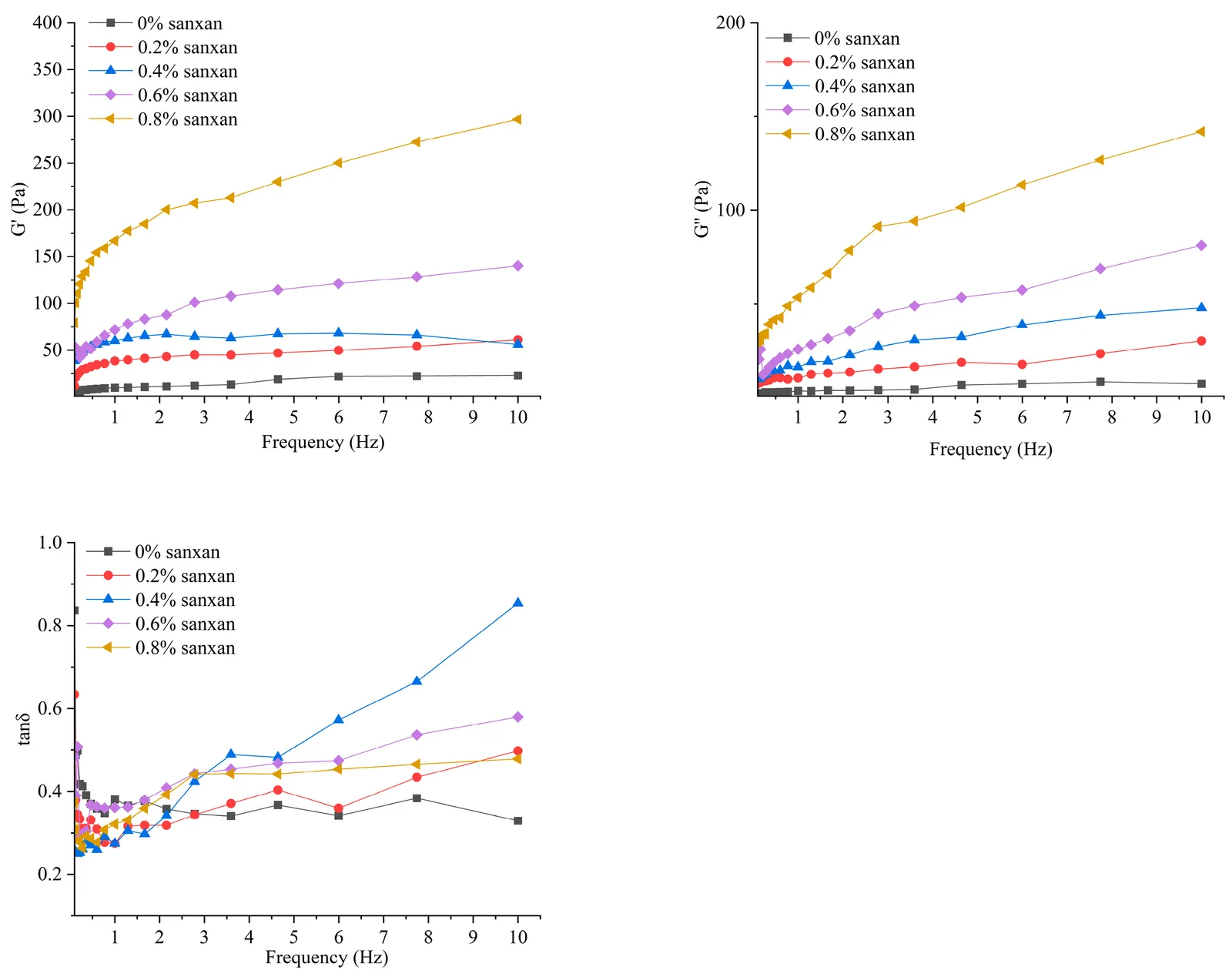
Effect of sanxan gum on rheological properties of coating batter.

Sanxan gum markedly enhanced the viscoelastic properties of rice flour-based coating batter in a concentration-dependent manner (Figure 2). Across the tested frequency range (0.1–10 Hz), all batter samples exhibited storage modulus (*G*′) > loss modulus (*G*″) (tan *δ* < 1), indicating predominantly elastic, solid-like behavior, consistent with other gluten-free batter systems supplemented with hydrocolloids. Both *G*′ and *G*″ increased progressively with rising sanxan gum addition amount. This reinforcement of the elastic network suggests that sanxan molecules interact with starch granules and leached amylose to form a cohesive, interconnected gel structure. This enhanced elasticity is expected to promote the formation of a uniform, continuous crust that effectively limits oil absorption during frying [7]. A similar dose-dependent increase in *G*′ has been reported for xanthan gum in rice-buckwheat batters [27]. However, this reinforcing effect contrasts with that reported for sanxan gum in *Cyperus esculentus* starch gels, where it decreased apparent viscosity, *G*′, and *G*″ [10], indicating that the influence of sanxan gum on rheological properties is also matrix-dependent, likely due to differences in starch source and the absence of gluten in the rice flour batter. The synergistic thickening between sanxan gum and rice starch may compensate for the structural deficiency inherent to gluten-free batters, consistent with the finding that sanxan gum enhances the rheological properties of gluten-free dough by promoting intermolecular cross-linking and increasing the viscosity of the continuous phase [28]. At sanxan gum addition amounts ≤ 0.6%, *G*′ was nearly independent of frequency over 0.1–10 Hz, indicating a relatively frequency-insensitive network. In contrast, at 0.8% sanxan gum, *G′* increased markedly with frequency, indicating stronger frequency dependence. This suggests a more frequency-dependent network with enhanced physical entanglement and structural heterogeneity, rather than a stronger homogeneous gel. The shift in frequency dependence indicates that a more entangled but less homogeneous network was formed at the highest sanxan gum addition amount. Although the absolute *G*′ and *G*″ values were highest at 0.8%, the increased frequency dependence and higher tan *δ* indicate that this high viscoelasticity originated mainly from physical entanglement and heterogeneous network structures, rather than from a uniform, strong gel network. Compared with conventional hydrocolloids such as xanthan gum and guar gum, which typically require higher concentrations to achieve comparable elasticity and often lead to excessive viscosity or higher tan *δ* values [29,30], sanxan gum produced a greater increase in *G*′ relative to *G*″, resulting in lower tan *δ* values and a more solid-like character at low concentrations (0.2–0.6%). At 0.8%, however, the increased tan *δ* and frequency dependence indicate a less uniform network with greater viscous contribution and structural discontinuity, suggesting that the network formed at this addition amount was more heterogeneous and less structurally uniform.

### 3.6. Apparent Viscosity and Batter Pick-Up of Coating Batter with Various Addition Amount of Sanxan Guan

**Figure 3:**
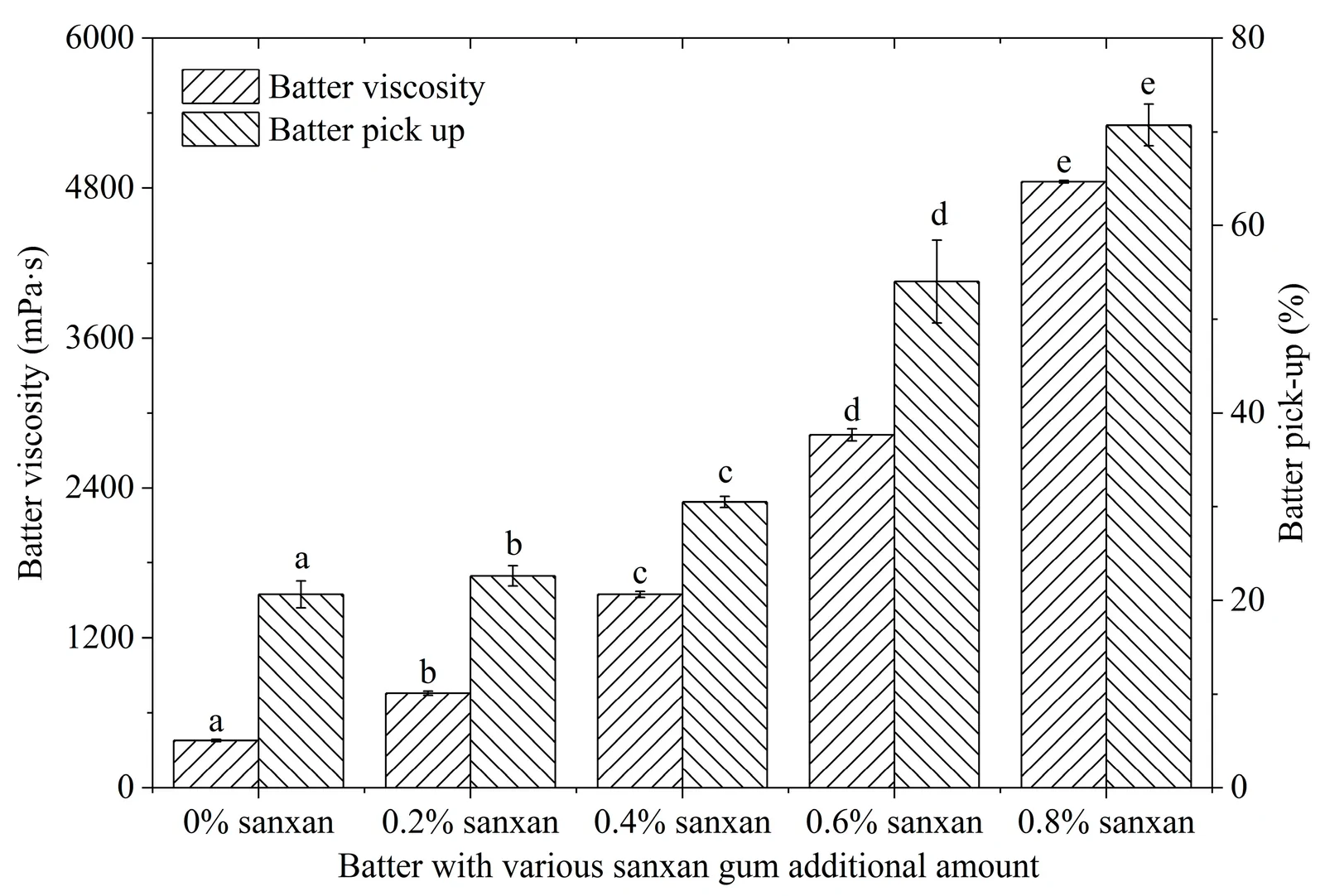
Effect of sanxan gum on apparent viscosity and batter pick-up of coating batter. Different lowercase letters above the bars indicate significant differences among samples with various sanxan gum addition levels (*p* < 0.05).

Apparent viscosity and batter pick-up increased significantly with increasing sanxan gum addition amount, and significant differences were observed among all addition levels (Figure 3). At 0.4% sanxan gum, the apparent viscosity already reached 1548 ± 24 mPa·s, indicating that a continuous network had formed. This concentration-dependent increase is consistent with the rheological behavior of sanxan gum solutions, in which the transition from a dilute solution to a semi-dilute entangled network leads to a marked increase in flow resistance [31]. Below this concentration, the rigid sanxan gum chains are largely dispersed and contribute little to flow resistance, whereas above it, chain overlap and entanglement restrict water mobility and sharply increase viscosity. Batter pick-up closely followed the apparent viscosity trend, consistent with previous reports on hydrocolloid-containing flour batters, in which apparent viscosity was positively correlated with batter adhesion [32,33]. These results indicate that the formation of an entangled gum network is the primary driver of improved coating adhesion. Balangu seed gum at 1.5% similarly increased pick-up compared with the control, but a further increase to 2% reduced pick-up because excessive viscosity hindered uniform application [18]. A comparable decline beyond an optimal viscosity was observed for xanthan gum and soybean fiber in battered fish nuggets [34]. In contrast, no such decline occurred up to 0.8% sanxan gum, which may be attributed to the pronounced shear-thinning behavior of sanxan gum solutions that lack thixotropy and time dependency [10]. Adequate stirring of the coating batter before use reduces its viscosity through shear thinning, allowing the batter to spread and adhere more uniformly even at high gum concentrations. These results demonstrate that sanxan gum effectively addresses the low viscosity and poor pick-up of rice flour-based coating batters, thereby improving their handling properties. Consequently, the concentration-dependent increase in batter pick-up resulted in a proportionally thicker coating layer, which critically modulates heat transfer and moisture evaporation during frying.

### 3.7. Effect of Sanxan Gum on Quality Parameters of Fried Crust and Meat

Sanxan gum addition amount markedly influenced the colour, texture, and moisture and oil contents of the fried products (Table 4). Meat tenderness improved as sanxan gum addition increased from 0% to 0.6%, with no further significant change at 0.8%. The crust oil content was higher at 0.4% than at lower addition amounts but then declined at 0.6% and 0.8%. Crust moisture remained stable up to 0.4% and increased thereafter, especially at 0.8%, whereas meat moisture increased continuously over the whole addition range. These trends indicate that the effects of sanxan gum on oil uptake and moisture retention are closely related to changes in coating thickness and crust structure. The low total oil content in the control and 0.2% samples (0.21–0.24 g/piece) is simply a result of their limited crust mass due to extremely low batter pick-up. Crucially, this thin crust failed to retain meat moisture, as evidenced by the markedly lower meat tenderness. According to the water-replacement mechanism [35], extensive moisture loss inherently creates pathways for oil ingress. Thus, the low oil content in these samples reflects a functionally inadequate coating rather than a superior oil-barrier performance. As the sanxan gum addition amount increased to 0.4%, the crust oil content was higher, which can be attributed to the increased batter pick-up and coating thickness. However, the meat moisture content increased substantially, and meat tenderness improved, suggesting that the thicker crust began to provide thermal protection and moisture retention for the meat. At 0.6% sanxan gum, the oil content decreased significantly compared with 0.4%, accompanied by the highest meat tenderness. Although the total oil content per piece was higher than that at 0.4% due to the increased batter pick-up required for thermal protection, this structural adjustment ultimately achieved an optimal balance between oil-barrier performance and moisture retention. The moderate batter viscosity and pick-up at this concentration produced a coating layer of appropriate thickness that restricted both moisture loss from the meat and oil penetration into the crust. This is consistent with previous findings that an optimal hydrocolloid concentration improves gel network integrity and reduces both moisture loss and oil uptake [26]. A further decrease in crust oil content was observed at 0.8% sanxan gum addition, accompanied by a marked increase in crust moisture content and a significantly increased crust thickness. However, the pronounced moisture retention and excessive crust mass at 0.8% fundamentally alter the compositional proportions, exerting a dilution effect on the oil content. The decreased oil content is primarily a compositional consequence rather than a true enhancement of oil-barrier capacity. Therefore, the total oil content per piece was evaluated. Results showed that 0.8% and 0.6% samples exhibited statistically equivalent total oil per piece (0.69 g and 0.70 g, respectively, *p* > 0.05). Combined with the absence of a significant difference in meat tenderness, it can be concluded that increasing the concentration beyond 0.6% did not confer additional practical benefits. This lack of practical improvement at 0.8% is attributed to the detrimental effects of the excessively thick coating. As the batter viscosity and pick-up increased, the dense coating substantially impeded heat transfer and moisture evaporation. From a practical processing perspective, this suggests that high-moisture coatings may require a prolonged frying time to achieve the desired crispy texture and an equivalent cooking endpoint. Furthermore, such a thick layer can lead to internal vapor pressure accumulation, causing structural rupture or the formation of closed pores. During cooling, these defects and closed pores draw surface oil into the crust through capillary action [36]. Excessive sanxan gum addition may also induce network inhomogeneity or partial phase separation, creating structural discontinuities that serve as preferential pathways for oil ingress [37]. This is consistent with the rheological results, in which the increased frequency dependence of *G′* and higher tan *δ* at 0.8% indicated greater network heterogeneity. Similar adverse effects of excessive hydrocolloid addition have been reported in deep-fat fried battered fish nuggets with xanthan gum and in deep-fried batter-coated meat strips with carboxymethyl cellulose [26,36]. These results indicate that although higher sanxan gum addition amounts further reduce crust oil content, they may introduce microstructural drawbacks that could compromise overall product quality.

In addition to texture and moisture, crust colour was also affected by sanxan gum addition. Increasing sanxan gum made the crust lighter and more yellow, as indicated by higher L* and b* values and a lower a* value. Specifically, the product prepared using the coating batter containing 0.6% sanxan gum exhibited a golden-brown crust, whereas at 0.8% addition the crust was distinctly paler. This colour change is related to the increased crust moisture content and the suppressed Maillard reaction. At higher addition amounts, the markedly increased crust moisture content diluted the concentration of surface reactants and may also have lowered the surface temperature during the early stage of frying, thereby inhibiting browning. This moisture-mediated suppression of browning implies that optimizing frying conditions, such as appropriately prolonging the frying duration, may be necessary to compensate for the high-moisture barrier when excessive hydrocolloids are applied, thereby ensuring a consistent golden-brown appearance. Similarly, Ma et al. [38] reported that in fried battered oysters, carboxymethyl cellulose enhanced the batter gel barrier and water-holding capacity, reduced moisture loss during frying, and significantly improved crust colour. The gel barrier effect was suggested to weaken the oxidative-Maillard coupling environment and thus inhibit the formation of brown products. Therefore, the colour change is an important manifestation of the structural and sensory drawbacks at higher addition levels.

### 3.8. Appearance and Microstructure of Crust with Various Addition Amount of Sanxan Guan

**Figure 4:**
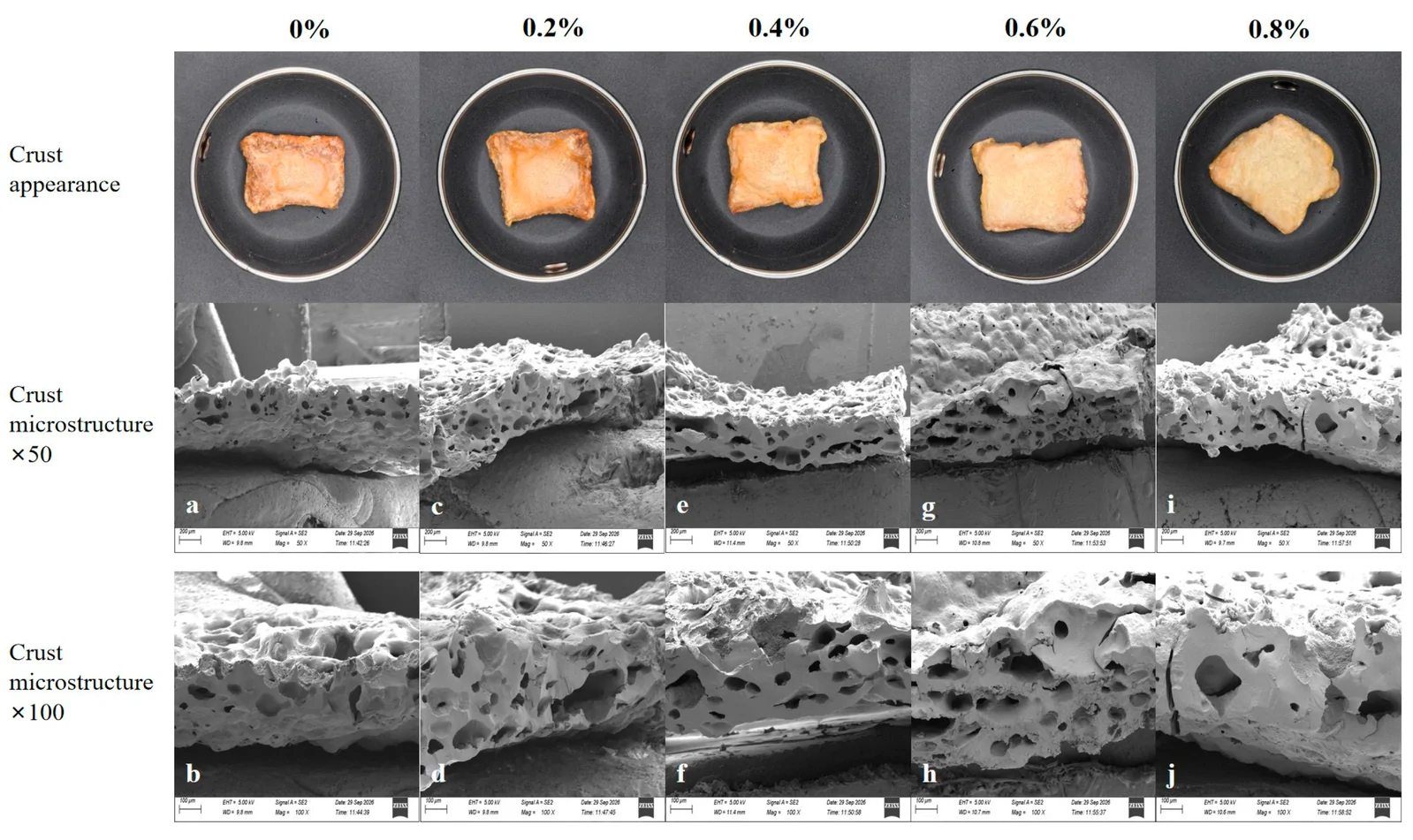
Effect of sanxan gum on the crust appearance and microstructure. (**a**–**j**) represent the microstructure of the crust of composite flour containing 0%, 0.2%, 0.4%, 0.6%, and 0.8% sanxan gum, observed at magnifications of 50× and 100×, respectively.

The crust appearance and SEM images revealed that both the thickness and microstructure of the fried crust were markedly affected by sanxan gum addition. As can be seen from the crust appearance, a markedly thinner crust was formed in the control and 0.2% samples than in those containing higher amounts of sanxan gum. The thinner crusts of these samples, resulting from their lower batter pick-up, underwent more rapid dehydration and setting during frying, causing water vapour to escape through fewer channels that coalesced into large pores before structural fixation. In the absence of a continuous gel network, pore-wall stabilization was weakened, resulting in a heterogeneous pore distribution. Consequently, the control and 0.2% crusts exhibited fewer but larger pores with irregular shapes and a heterogeneous distribution (Figure 4a–d). These morphological characteristics are highly consistent with previous findings that batter thickness and water evaporation rate strongly influence pore development in fried coatings [14,39]. Such an open and heterogeneous architecture readily permits rapid moisture loss, which directly accounts for the reduced meat moisture and tenderness observed in these samples. With sanxan gum addition, the crust thickness increased, attributed to the enhanced viscosity of the batter that promoted greater pick-up and a more uniform coating layer. At 0.4% sanxan gum (Figure 4e,f), the pore size decreased, and the pore distribution became more uniform relative to the control and 0.2% samples. However, the crust oil content was higher, which can be attributed to the increased batter pick-up and coating thickness. The meat moisture content and tenderness improved, indicating that the thicker crust began to provide thermal protection and moisture retention for the meat. At 0.6% sanxan gum addition (Figure 4g,h), the crust exhibited a more continuous and homogeneous gel network with smaller and more evenly distributed pores. This continuous network matrix explains the significantly lower oil content and improved meat tenderness compared with 0.4%, despite a higher total oil content per piece. The intermediate batter viscosity and pick-up at 0.6% sanxan gum addition yielded a coating of suitable thickness that simultaneously maintained barrier integrity and structural uniformity. This interpretation is supported by the recovery of Δ*H* in the DSC results and by the relatively frequency-independent *G*′ with low tan *δ* in the rheological results at 0.6% sanxan gum addition. At 0.8% sanxan gum addition (Figure 4i,j), the crust became thicker, and the microstructure deteriorated markedly, exhibiting larger pores, cavities, cracks, and a highly heterogeneous distribution. These structural discontinuities likely disrupted the continuity of the oil-barrier layer and created channels for oil penetration. The excessive coating thickness may also have impeded heat transfer and moisture evaporation, leading to internal vapour pressure accumulation and structural rupture. During cooling, the resulting defects and closed pores can draw surface oil into the crust through capillary action [35,36]. Such microstructural features are consistent with the increased frequency dependence of *G*′ and higher tan *δ* at 0.8% sanxan gum addition, indicating greater network heterogeneity and partial phase separation. Consequently, the structural damage observed here offset the theoretical barrier advantages of a thicker coating, leading to no practical improvement in oil reduction or tenderness compared to 0.6%. Thus, the functionality of the batter coating depends on the balance between coating thickness and network continuity, rather than on thickness alone. Moreover, a recent review explicitly states that various polysaccharide complexes are more effective in reducing oil uptake in fried foods [40], with xanthan gum-guar gum blends outperforming single-gum systems in reducing the oil uptake of fried carrot slices. This suggests that the structural drawbacks of excessive single-gum addition might be mitigated through synergistic hydrocolloid blending, which warrants further investigation.

## 4. Conclusions

This study investigated the application of sanxan gum in a rice flour-based coating batter to address the low viscosity and weak structural integrity arising from the absence of gluten, with the aim of improving batter handling properties and reducing oil uptake during deep-fat frying. Sanxan gum increased batter viscosity and pick-up and restricted starch granule swelling through competitive hydration. At 0.6% addition, the crust oil content decreased and meat tenderness and moisture content improved, while a continuous and homogeneous gel network was formed. Although the 0.8% addition showed the lowest crust oil percentage, its total oil content per piece was equivalent to that of 0.6%, and the microstructure deteriorated with network inhomogeneity without further improving meat tenderness. This confirms that 0.6% addition achieves the optimal balance between practical oil reduction and overall quality. LF-NMR results showed that the oil-reducing effect of sanxan gum was not mediated by changes in water mobility. It was mainly associated with increased batter viscosity and restricted starch swelling and with the formation of a continuous gel network. These results indicated that sanxan gum has potential as a functional hydrocolloid for improving the quality of rice flour-based fried products. However, precise control over its incorporation level is required to prevent structural defects. Moreover, because high concentrations of sanxan gum significantly impede moisture evaporation, future industrial applications and studies should consider optimizing dynamic frying parameters (e.g., adjusting frying time based on coating thickness) to achieve consistent textural and sensory endpoints. Therefore, future work may explore blending sanxan gum with other hydrocolloids to achieve synergistic oil reduction and quality improvement, and a comprehensive sensory evaluation should be conducted once sanxan gum is officially approved for fried battered products.

## Figures and Tables

**Table 1:** Effect of sanxan gum on the hydration properties of rice flour.

Samples	WHC ^1^	OAC	WAI	WSI	SP
0%	0.89 ± 0.00 ^c^	1.07 ± 0.01 ^b^	6.62 ± 0.00 ^a^	2.99 ± 0.28 ^c^	6.83 ± 0.02 ^a^
0.2%	0.90 ± 0.01 ^c^	1.08 ± 0.02 ^b^	6.44 ± 0.14 ^ab^	3.17 ± 0.06 ^bc^	6.65 ± 0.14 ^ab^
0.4%	0.87 ± 0.02 ^c^	1.01 ± 0.07 ^b^	6.27 ± 0.05 ^bc^	4.54 ± 0.86 ^a^	6.57 ± 0.01 ^c^
0.6%	0.93 ± 0.00 ^b^	1.15 ± 0.09 ^ab^	6.17 ± 0.13 ^c^	4.08 ± 0.04 ^ab^	6.43 ± 0.14 ^bc^
0.8%	0.95 ± 0.01 ^a^	1.27 ± 0.07 ^a^	6.28 ± 0.08 ^bc^	3.96 ± 0.11 ^ab^	6.54 ± 0.11 ^c^

^1^ WHC: Water-holding capacity; OAC: oil-absorption capacity; WAI: water absorption index; WSI: water solubility index; SP: swelling power. Different superscript letters within the same column indicate significant differences (*p* < 0.05).

**Table 2:** Effect of sanxan gum on pasting properties of rice flour.

Samples	Peak Viscosity (cP)	Trough Viscosity (cP)	Break Down (cP)	Final Viscosity (cP)	Set Back (cP)	Peak Time (min)	Pasting Temperature (°C)
0%	2966.33 ± 64.76 ^a,1^	2346.00 ± 16.64 ^a^	620.33 ± 55.22 ^c^	5307.00 ± 274.86 ^b^	2961.00 ± 267.42 ^c^	6.49 ± 0.10 ^a^	87.78 ± 0.85 ^a^
0.2%	2842.33 ± 122.26 ^a^	2359.67 ± 91.30 ^a^	482.67 ± 32.65 ^b^	5019.00 ± 202.24 ^ab^	2659.33 ± 106.75 ^b^	6.51 ± 0.03 ^a^	87.48 ± 0.52 ^a^
0.4%	2916.00 ± 41.01 ^a^	2535.50 ± 55.86 ^ab^	380.50 ± 14.85 ^ab^	4996.00 ± 55.15 a^b^	2460.50 ± 0.71 ^b^	6.73 ± 0.00 ^b^	88.00 ± 0.00 ^b^
0.6%	2830.33 ± 100.08 ^a^	2558.00 ± 84.79 ^ab^	272.33 ± 18.77 ^a^	4746.33 ± 141.89 ^a^	2188.33 ± 72.45 ^a^	6.96 ± 0.07 ^c^	87.75 ± 0.48 ^ab^
0.8%	2913.00 ± 84.85 ^a^	2647.00 ± 72.12 ^b^	266.00 ± 12.73 ^a^	4848.00 ± 152.74 ^ab^	2201.00 ± 80.61 ^a^	7.00 ± 0.00 ^c^	87.18 ± 0.04 ^a^

^1^ Different superscript letters within the same column indicate significant differences (*p* < 0.05).

**Table 3:** Effect of sanxan gum on starch gelatinization parameters of coating batter.

Sample	*T*_o_/°C	*T*_p_/°C	*T*_c_/°C	Δ*H*/(J/g)
0% sanxan	66.96 ± 0.18 ^b,1^	71.65 ± 0.29 ^b^	76.74 ± 0.29 ^b^	0.74 ± 0.01 ^a^
0.2% sanxan	67.35 ± 0.03 ^ab^	71.93 ± 0.20 ^ab^	77.04 ± 0.72 ^ab^	0.57 ± 0.01 ^c^
0.4% sanxan	67.34 ± 0.17 ^ab^	72.15 ± 0.25 ^ab^	77.83 ± 0.05 ^a^	0.55 ± 0.01 ^c^
0.6% sanxan	67.45 ± 0.24 ^a^	72.40 ± 0.28 ^a^	77.62 ± 0.20 ^ab^	0.66 ± 0.01 ^b^
0.8% sanxan	67.27 ± 0.01 ^ab^	71.97 ± 0.08 ^ab^	77.78 ± 0.25 ^a^	0.73 ± 0.00 ^a^

^1^ Different superscript letters within the same column indicate significant differences (*p* < 0.05).

**Table 4:** Effects of sanxan gum on quality parameters of fried crust and meat.

Sample	Fried Crust						Meat	
	Moisture Content (%)	Oil Content (%)	Total Oil Content per Piece (g)	L*	a*	b*	Tenderness (N)	Moisture Content (%)
0% sanxan	19.63 ± 0.75 ^a,1^	18.44 ± 0.03 ^b^	0.21 ± 0.02 ^a^	62.07± 3.08 ^a^	4.56 ± 0.90 ^c^	12.73 ± 0.73 ^a^	58.18 ± 6.96 ^c^	37.42 ± 0.54 ^a^
0.2% sanxan	18.86 ± 0.74 ^a^	19.50 ± 0.14 ^c^	0.24 ± 0.01 ^a^	66.70 ± 2.21 ^b^	5.30 ± 0.70 ^c^	17.95 ± 1.60 ^b^	56.14 ± 1.53 ^c^	42.55 ± 0.38 ^b^
0.4% sanxan	19.56 ± 0.49 ^a^	23.43 ± 0.41 ^e^	0.43 ± 0.01 ^b^	71.56 ± 2.80 ^c^	4.77 ± 0.63 ^c^	22.61 ± 1.89 ^c^	42.24 ± 1.27 ^b^	48.50 ± 0.59 ^c^
0.6% sanxan	20.80 ± 0.52 ^b^	21.33 ± 0.21 ^d^	0.70 ± 0.06 ^c^	74.67 ± 2.31 ^d^	2.20 ± 0.58 ^b^	23.70 ± 1.60 ^c^	25.11 ± 2.39 ^a^	56.19 ± 0.21 ^d^
0.8% sanxan	31.65 ± 0.56 ^c^	15.07 ± 0.26 ^a^	0.69 ± 0.03 ^c^	81.23 ± 1.50 ^e^	−0.16 ± 0.30 ^a^	26.14 ± 1.89 ^d^	24.05 ± 2.72 ^a^	64.61 ± 1.19 ^e^

^1^ Different superscript letters within the same column indicate significant differences (*p* < 0.05).

## Data Availability

The original contributions presented in this study are included in the article. Further inquiries can be directed to the corresponding author.

## Abbreviations

The following abbreviations are used in this manuscript:

WHC	Water-holding capacity
OAC	Oil-absorption capacity
WAI	Water absorption index
WSI	Water solubility index
SP	Swelling power
RVA	Rapid visco analyzer
LF-NMR	Low-field nuclear magnetic resonance
DSC	Differential scanning calorimetry
*T*_o_	Onset temperature
*T*_p_	Peak temperature
*T*_c_	Conclusion temperature
Δ*H*	Gelatinization enthalpy
*G*’	Storage modulus
*G*”	Loss modulus
tan *δ*	Loss tangent
*T*_2_	Transverse relaxation time
SEM	Scanning electron microscope
